# Shift Work or Food Intake during the Rest Phase Promotes Metabolic Disruption and Desynchrony of Liver Genes in Male Rats

**DOI:** 10.1371/journal.pone.0060052

**Published:** 2013-04-02

**Authors:** Roberto C. Salgado-Delgado, Nadia Saderi, María del Carmen Basualdo, Natali N. Guerrero-Vargas, Carolina Escobar, Ruud M. Buijs

**Affiliations:** 1 Departamento de Biología Celular y Fisiología, Instituto de Investigaciones Biomédicas, Universidad Nacional Autónoma de México, Distrito Federal, México; 2 Departamento de Biología Celular, Facultad de Ciencias, Universidad Autónoma de San Luis Potosí, San Luis Potosí., México; 3 Departamento de Anatomía, Facultad de Medicina, Universidad Nacional Autónoma de México, Distrito Federal, México; Vanderbilt University, United States of America

## Abstract

In the liver, clock genes are proposed to drive metabolic rhythms. These gene rhythms are driven by the suprachiasmatic nucleus (SCN) mainly by food intake and via autonomic and hormonal pathways. Forced activity during the normal rest phase, induces also food intake, thus neglecting the signals of the SCN, leading to conflicting time signals to target tissues of the SCN. The present study explored in a rodent model of night-work the influence of food during the normal sleep period on the synchrony of gene expression between clock genes and metabolic genes in the liver. Male Wistar rats were exposed to forced activity for 8 h either during the rest phase (day) or during the active phase (night) by using a slow rotating wheel. In this shift work model food intake shifts spontaneously to the forced activity period, therefore the influence of food alone without induced activity was tested in other groups of animals that were fed *ad libitum*, or fed during their rest or active phase. Rats forced to be active and/or eating during their rest phase, inverted their daily peak of Per1, Bmal1 and Clock and lost the rhythm of Per2 in the liver, moreover NAMPT and metabolic genes such as *Pparα* lost their rhythm and thus their synchrony with clock genes. We conclude that shift work or food intake in the rest phase leads to desynchronization within the liver, characterized by misaligned temporal patterns of clock genes and metabolic genes. This may be the cause of the development of the metabolic syndrome and obesity in individuals engaged in shift work.

## Introduction

The suprachiasmatic nucleus in the hypothalamus (SCN) generates and transmits circadian rhythms to other neuronal structures. Circadian rhythms within cells are driven by a series of interacting clock genes (*Per, Cry, Clock and Bmal1*) resulting in 24 hour cycles of gene expression [1]–[3]. Via autonomic and hormonal pathways as well as via temporal patterns in food intake and body temperature, the SCN drives the rhythmic expression of clock genes in peripheral tissues [4]–[7] and thus maintains physiological rhythms coupled to the light/dark (LD) cycle.

Clock genes may interact with metabolic genes and regulate their transcription, endowing cells and tissues with rhythmic molecular mechanisms that allow adapting to the daily cycles in metabolic demand [8]–[13]. Especially genes that regulate gluconeogenesis and fatty acid oxidation are suggested to interact with clock genes for their rhythmic expression [9], [13]. This interaction has been demonstrated in vitro for genes that are regulators of metabolic functions in liver cells, such as the peroxisome proliferator-activated receptors (PPARs) like Pparγ, Pparα, Pparβ, Pgc-α1 and silent mating-type information regulator homolog1 (Sirt1) member of the Sirtuin family.

Decreased expression of both Sirt1 and Pparγ is associated with development of the metabolic syndrome [14]–[15]. Likewise changes in metabolic condition, especially the redox state and energy availability, influence the expression of Bmal1 and Per2, suggesting a strong interaction between metabolic and circadian regulation in liver cells [16], [17]. Consequently a disturbed interaction between clock genes and metabolic genes is proposed to be the molecular basis of metabolic disruption due to circadian desynchrony [18], [19].

Circadian disruption is induced by night- and shift-work, which in the long term lead to overweight, increased abdominal fat deposition and development of indicators of metabolic syndrome [20]–[21]. Using a rat model of shift work, we have demonstrated that forced activity in slowly rotating drums during the rest phase induces a circadian misalignment based on a spontaneous temporal shift of food intake, of metabolic parameters and a loss in blood glucose rhythm, while hormonal rhythms, predominantly dependent on the SCN, remained unchanged. This could be concluded since shift-working animals that were not able to eat but only had food access in their active phase, did not develop this circadian misalignment [22]. In contrast, rats exposed to forced activity during the active phase, do not show signs of circadian disruption. This scheduled activity protocol was not perceived as stressful since corticosterone levels were hardly changed [22]–[23]. These observations suggest that activity during the rest phase and the resulting inverted feeding patterns cause a loss of synchrony between the SCN and the liver which is mainly driven by metabolic signals and by autonomic and hormonal time signals driven by the SCN [22]–[25]. Although several studies have shown that the time of feeding affects metabolism and clock gene expression in the liver [24], [25], this is the first study to measure metabolic parameters and gene expression rhythms in the shift work rat model the authors’ group has developed. Moreover in view of the fact that NAMPT plays a critical role in a number of biological processes through the NAD+ dependent deacetylase SIRT1 [26]; we focused in the present study to analyze in our shift-work model this relationship between clock gene expression and the NAMPT-NAD-SIRT1 cascade including the measurement of Pparα, Pparγ and Pgc1α. We hypothesized that an altered activity pattern combined with a shifted feeding pattern may lead to loss of synchrony between clock genes and metabolic genes in the liver and especially affect this cascade.

Circadian misalignment was induced by placing rats in a slow rotating wheel for 8 hours during their rest phase (ARP), this was compared with rats placed in a slow rotating wheel during their active phase (AAP) and with undisturbed controls. Another set of rats was exclusively exposed to restricted food intake in the rest period (FRP) or in the active period (FAP). We demonstrate that food intake alone (FRP) or activity combined with food intake (ARP) during the rest phase induce a desynchronization in the liver between clock genes and metabolic genes, being a possible cause for metabolic disruptions associated with circadian misalignment.

## Materials and Methods

### Animals and Housing

Male Wistar rats weighing 140–170 g at the beginning of the experiment (4–5 weeks old) were obtained from the animal facility of the Faculty of Medicine of the UNAM that uses a rotational breeding system to obtain out bred Wistar rats. The animals were housed in individual transparent acrylic cages (40×50×20 cm) placed in isolated lockers housing eight animals each in a soundproof room. Rats were maintained in a 12 h light, 12 h dark cycle, lights on defined as Zeitgeber Time 0 (ZT0), constant temperature (22+/−1°C), circulating air, and free access to water and food (Rodent Laboratory Chow 5001, IL) unless otherwise stated. Experiments were approved by the committee for ethical evaluation at the Universidad Nacional Autónoma de Mexico, in strict accordance with the Mexican norms for animal handling, Norma Oficial Mexicana NOM-062-ZOO-1999.

### Experimental Design

For a baseline all rats were monitored in their home cages for 8–10 days in LD conditions. A first series of rats (N = 54) was randomly assigned to Control or forced activity conditions. Control rats (CTRL, n = 18) were housed in individual cages in the monitoring system and left undisturbed during the protocol except for cleaning the cages and implantation of the jugular cannula. The forced-activity rats were subdivided in two groups: 1- activity during the normal resting phase (ARP; *n* = 18), 2- activity during the normal active phase (AAP, n = 18). CTRL and ARP rats were maintained in a regular 12 h LD cycle with lights on at 07∶00 h, while rats for the AAP group were kept in an inverted 12 h LD cycle with lights on at 1900 h for 12–15 days in DL condition before starting the protocol.

A second series of rats (n = 36) was assigned to restricted food access in the home cage. One group (n = 18) had exclusively food access during the normal rest phase (FRP) from ZT0–ZT12 and a second group (N = 18) had access to food during the normal active phase (FAP) from ZT12–ZT0. This food restriction procedure as well as the compulsory protocol was carried out for 5 weeks.

### Forced Activity Protocol

Rats were placed 8 h daily in slow rotating wheels that are used for sleep deprivation (33 cm diameter×33 cm wide) with four concentric subdivisions, which allows individual housing of four rats. Drums rotate with a speed of one revolution/3 min and force rats to stay awake. Due to the slow movement of the wheels, rats do not need to walk; they can sit, groom, and even lie down. In addition, they can drink and eat freely from a small bottle and pellets hanging from the middle tube [see [23] for more details and a video of the rat activity].

Starting on a Monday ARP and AAP rats were placed in the rotating drums for 8 hours from 0900–1700 geographical time, which represented for ARP rats ZT2–ZT10 and for AAP rats ZT14–ZT22. For this last group, maintained in an inverted cycle, the slow rotating wheel was placed in an enclosed chamber maintained in darkness. Rats were transferred to and from the wheel with a safe red light.

After 8 h in the drums, rats were returned to their home cages and remained undisturbed until the next day. This procedure was carried out for 5 weeks from Monday to Friday. During weekends all rats remained undisturbed in their home cages.

### Body Weight and Food Intake

Rats were weighed before starting baseline and at the end of the 4th week of activity or feeding manipulations. Body weight gain was calculated for this interval and for each group. Ingested food was monitored and weighed before starting the feeding protocol and in the fourth week of the protocol by weighing separately the nocturnal and the diurnal consumption (n = 8 per group).

Ingested food was monitored twice every week during the baseline period and during the four working weeks by weighing separately the nocturnal and the diurnal consumption and the consumption in the wheels for the ARP and AAP rats.

Rats assigned to the *ad libitum* (CTRL) group had always free access to food in their home cages as well as the animals in the rotating drums (ARP and AAP). Rats assigned to the group with restricted food during the rest period (FRP) had access to food from ZT0–ZT12; therefore food was removed from the feeder at the time of lights off and replaced in the feeder after lights on, and the group with restricted food during the activity period (FAP), had access to food from ZT12–ZT24; and the food was removed from the feeder at the time of light on and replaced at lights off.

### Intraperitoneal Glucose Tolerance Test (GTT)

On the Friday of the fourth experimental week, rats underwent surgery to implant a jugular cannula as previously described [22]. After one recovery weekend the animals were subjected to the same working.

or feeding protocol and at the end of the 5th week the CTRL, ARP, AAP and FRP rats were fasted overnight and the FAP rats were fed during the first 4 hours in the active phase and subsequently food was removed. The following day, after 16 h or 12 h fasting, all animals were tested 4 hours after light onset, in order that all animals were tested at the same circadian time (ZT 4). 1 g of glucose/kg body weight in saline solution (1.1 ml) was injected i.p in all groups and blood samples (50 ul) were collected from the cannula before glucose administration, and 15, 30, 60, 90 and 120 min after glucose administration. Glucose level was determined with a blood glucose monitor (Glucose meter, Optium Xceed. Chip, Abbott).

### Tissue Collection

At the end of the fifth working week, the rats were randomly assigned to one of six temporal points (0, 4, 8, 12, 16, 20 hours after light on) to complete a 24 h cycle in order to determine rhythmicity of various genes and proteins (*N* = 3 per temporal point). Rats were anesthetized with an overdose of sodium pentobarbital (Sedal-Vet 65 mg/ml), and part of the left lobule of the liver was quickly removed and immediately frozen at −80°C while another part was placed in 4% paraformaldehyde (PFA). The abdominal fat pads were dissected and weighed.

### Oil-Red-O Staining

After post fixation of the liver for 24 h, it was cryoprotected in 30% sucrose for 3–4 days. Livers were frozen and cut in sections of 14 μ at −18°C, mounted and stained with Oil-Red-O (Sigma, St. Louis, MO, USA), which detects hydrophobic lipids, including esterified cholesterol. The sections were counterstained with Mayer’s hematoxylin (Sigma) and cover-slipped with a water-based resin. Three photographs of different areas of three representative sections of each animal were taken and the red staining was outlined within the photo using image J program.

### NAD^+^/NADH Measurements

The NAD+ in the livers was determined with a commercial kit for ELISA (NAD+/NADH Quantification Kit; BioVision Research Products, Mountain View, CA 94043 USA), according to the manufacturer’s instruction.

### Semi-quantitative RT- PCR

Total RNA was isolated from liver with Trizol (Invitrogen) according to the manufacturer’s recommendations. Equal quantities of total RNA from at least three rats per time point were used for complementary DNA generated by SuperScript III (Invitrogen). All metabolic genes *Nampt, Pparγ, Pparα, Sirt1, Pgc1α* and clock genes, *Bmal1, Clock, Per1 and Per2* were analyzed by quantitative reverse-transcriptase-mediated PCR (Q-RT–PCR). The oligonucleotide primers sequences are listed in Table 1. All data were normalized to *β*-*actin* expression. Expression of mRNA was quantified using a Bio-Rad Personal Molecular Imager FX, and quantification was performed using Scion Image for windows 4.0.

**Table 1:** Primer sequences for quantitative PCR.

GENE NAME		SEQUENCES	ACCESSION NUMBER	
Per1		Sense 5′-TGTGTTTCGGGGTGCTCGCT-3′	NM_001034125.1	
		Antisense 5′-CGTGCGCACTTTATGGCGGC-3′		
Per2		Sense 5′-TGCTGTGGCTGTGTCCCTGG	NM_031678.1	
		Antisense 5′-GGGACCGCCCTTTCGTCCAC		
Clock		Sense 5′-TTGATGGATTGGTGGAAGAAG	NM_021856	
		Antisense 5′-TCCTCGAAGCATGTGACAAC		
Bmal 1		Sense 5′-GGACTTCGCCTCTACCTGTTCA	NM_024362.2	
		Antisense 5′-AACCATGTGCGAGTGCAGGCGC		
Sirt 1		Sense 5′-TGACTGGACTCCAAGGCCACG	NM_001107627.1	
		Antisense 5′-CCCACAGGAAACAGAAACCCCAGC		
Pgc1α		Sense 5′-TGTGAATGACCTGGACACAGACAGC	NM_031347.1	
		Antisense 5′-TCCTGTGGGTGTGGTTTGCATGG		
Pparγ		Sense 5′-TGACCAGGCTGAGGGGACGG	NM_001145367.1	
		Antisense 5′-ATAAGGCGGGGACGCAGGCT		
Pparα		Sense 5′-GCGTAACTCACCGGGAGGCG	NM_013196.1	
		Antisense 5′-GAGCCCTCCGAGCCTGGACA		
Nampt		Sense 5′-GTGCTACTGGCTCACCAACTGGA	NM_342244.4	
		Antisense 5′-GCTGACCACAGACACAGGCACT		
b-Actina		Sense 5′- ATCGTGGGCCGCCCTAGGCA	NM_031144.2	
		Antisense 5′-ACGTACATGGCTGGGGTGTTG		

### Western Blot Analysis

Proteins were extracted from homogenized liver samples with a lysis buffer (200 mM NaOH, 1% w/v SDS) supplemented with protease inhibitor (pepstatin A, 0.1 mg aprotinin, 35 mg PMSF/ml, 1 mM de TPCK; all from Sigma–Aldrich) and phosphatase inhibitors (Phosphatase Inhibitor Mixture I; Sigma–Aldrich), using a TissueMiser homogenizer (Fisher Scientific) and clarified by centrifugation. Total liver protein (30 mg/ml) was separated on 12% Tris-glycine acrylamide gels and wet transferred to Hybond-C membranes, (Amersham Biosciences). Antibodies against SIRT1 (1∶200; Santa Cruz Biotechnology) and with donkey peroxidase-conjugated secondary antibody (Santa Cruz). Bands were detected by chemiluminescence using ECL plus Western blotting detection system (Amersham Pharmacia Biotech, Buckinghamshire, UK) following the manufacturer’s instructions. Anti-tubulin antibody (1∶750; Sigma–Aldrich) was used as an internal control.

### Statistical Analysis

Data were classified by groups and time and are represented as mean ± standard error of the mean (S.E.M.). Body weight gain, abdominal fat and red oil staining were analyzed with a one-way ANOVA for the main factor group. The GTT data were evaluated with a two-way ANOVA for the factors group (three levels) and time as a factor of repeated measures (six levels), this was followed by a Tukey multiple comparisons *post hoc* test with α set at *P<*0.05. Statistical analysis was performed with the program STATISTICA for Windows version 4.5 (StatSoft, 1993). Daily curves for clock and metabolic genes were tested with a cosinor analysis to determine amplitude and time of the acrophase significant amplitude was considered as P<0.05. The significant changes in time were corroborated with a Kruskall-Wallis test applied to each individual curve where the significant P value was set in P<0.05, this analysis was performed with Matlab and STATISTICA for Windows respectively.

## Results

### Compulsory Activity and Feeding during the Rest Phase Promote Increased Body Weight and Glucose Intolerance

**Figure 1:**
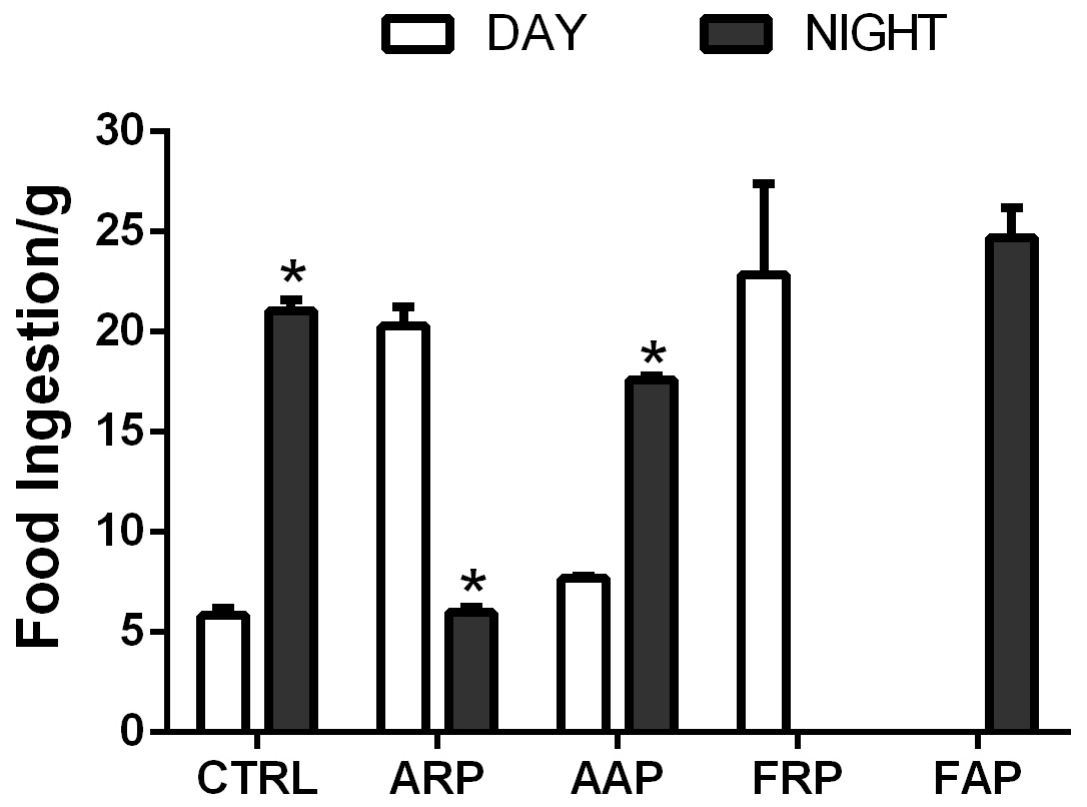
Mean food intake and S.E.M. of control, ARP, AAP, FRP and FAP rats during the day (white bars) and during the night (gray bars) during the fourth week of manipulation. Asterisks indicate statistical difference between day and night values (*P<*0.05).

**Figure 2:**
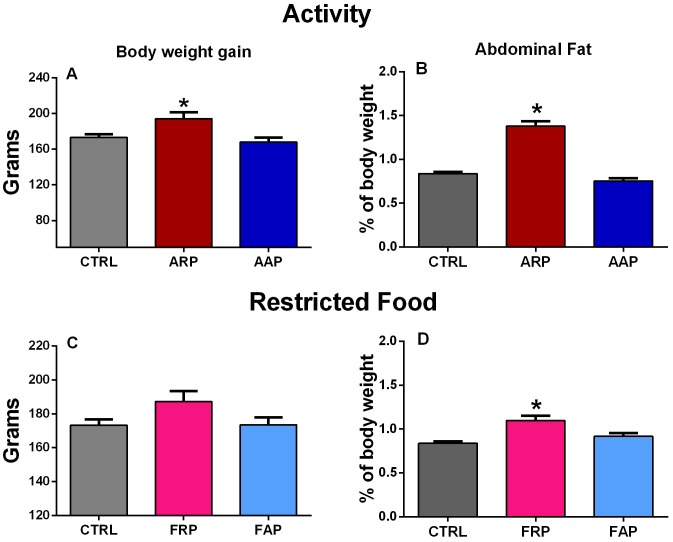
Changes in metabolic parameters after 4 weeks activity in the rest phase. Mean (±SEM) body weight gain (A,C), and abdominal fat weight (B,D) of control (n = 8) and rats exposed to forced activity during their rest phase (ARP) or during their activity phase (AAP) (n = 6 per group) and rats with restricted food during the rest phase (FRP) or during their activity phase (FAP) (n = 9 per group). All groups showed a similar daily pattern of food consumption); however, ARP and FRP rats increased their body weight (A,C) and attained a higher accumulation of abdominal fat (B,C) while AAP rats remained at similar values as controls. (P<0.001). Statistical analyses were performed with a Tukey multiple comparisons post hoc test.

During the last week of the experiment control and AAP rats ingested between 70–80% of their food during the active phase while ARP rats shifted their nocturnal pattern of food intake towards the activity hours resulting in 75–80% of food ingestion during the inactive phase (Fig. 1). Animals that were active during their rest phase consumed nearly all their food in the wheels, while animals that were placed in the wheels in their active phase also consumed food in the time before and after their wheel activity. In spite of a similar amount of food intake as *the ad libitum* controls and the AAP groups (26.14±0.76 g/day), ARP rats gained more body weight (Fig. 2A). The one way ANOVA indicated a significant difference among groups (F_(2,21)_ = 8.50; p<0.002) and the *post hoc* test confirmed a significant increase of the ARP group. The accumulation of abdominal fat was significantly increased in ARP as compared with CTRL and AAP animals, the ANOVA indicated a significant difference among groups (F_(2,21)_ = 83.31, p<0.0001; Fig. 2B) and the *pos hoc* test confirmed a significant increase for the ARP group. Rats exposed to scheduled access to food, ingested similar amounts as their controls (26.14±0.76 g per day). After 4 weeks the FRP group had a tendency for higher body weight than CTRL and FAP rats, however this did not reach statistical significance (F_(2,21)_ = 1.85; P = 0.063; Fig. 2C). Also, FRP accumulated more abdominal fat, than CTRL and FAP rats, which showed similar values (Fig. 2D). The one way ANOVA indicated a significant difference among groups (F_2,21)_ = 10.99p<0.001) and the *post hoc* test confirmed a significant increase of the FRP group. All these results were comparable as reported before [23].

**Figure 3:**
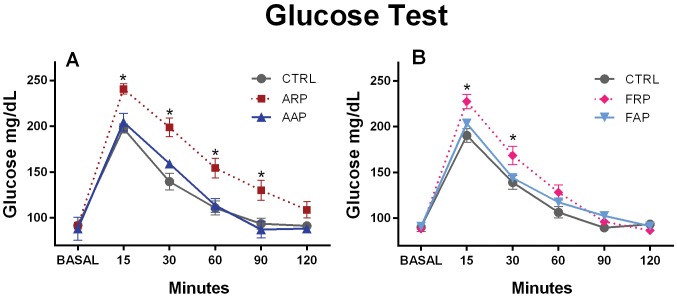
Glucose intolerance after 4 weeks of activity and or food in the rest phase. Animals received at time 0 (basal) an intraperitoneal injection of 1 g of glucose per kg body weight. Glucose levels in ARP animals show immediately after the glucose injection an increase in plasma glucose levels that remained significant higher as compared to the AAP and CTRL group until 90 minutes after the challenge. Glucose levels in FRP animals show after the glucose injection a similar increase in plasma glucose levels; however at 60 minutes no difference between FAP and CTRL group can be noted.

We performed a glucose tolerance test to investigate possible changes in insulin sensitivity and/or insulin secretion. After intraperitoneal injection of a glucose bolus, 15 min later all groups showed a peak in blood glucose, CTRL and AAP rats returned to normal values after 90 minutes while ARP rats maintained high glucose levels throughout the sampling period (Figure 3A). The two-way ANOVA indicated a significant difference between groups (F_(2,66)_ = 60.54; *P*<0.0001), a significant difference in time [F_(5,66) = _201.0; *P<*0.0001], and for the interaction of both factors [F_(10,66)_ = 3.77; *P<*0.0004]). Also the FRP animals showed significant higher glucose levels at 15 and 30 minutes after the glucose injection, as compared to their CTRL and FAP groups, however after 60 minutes they had lowered their glucose to similar values as the control. The two way ANOVA indicated a significant difference between groups (F_(2,72)_ = 13.99; *P*<0.0001), a significant difference in time (F_(5,72) = _277.33; *P<*0.0001), and for the interaction of both factors (F_(10,72)_ = 5.07; *P<*0.0001).

**Figure 4:**
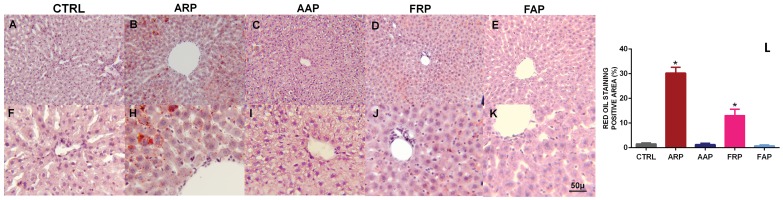
When rats are active and eat during the rest phase microvesicular steatosis were observed. Oil-Red-O staining of frozen sections of livers obtained from CTRL, ARP and AAP rats. Mayer’s hematoxylin was used for counterstaining. In ARP rats were observed typical lipid microdroplets that are indicative of microvesicular steatosis. In L is shown the mean of the area of the red oil positive tissues,; (*P<*0.0001).

In the CTRL and AAP livers few fat deposits were observed, while in the ARP rat numerous intracellular micro vesicular fat deposits were noted in the pericentral and midzonal areas of the hepatic lobules (Fig. 4). The lipid particles varied in size and appeared to be larger in the mid zonal areas compared to those in the central or peripheral areas and covered a significant higher area in the ARP and FRP rats (F_(4,35)_ = 66.23; *P<*0.0001).

### Compulsory Activity or Food Restricted to the Rest Phase Invert or Abolish Rhythmicity of Clock Genes in the Liver

**Figure 5:**
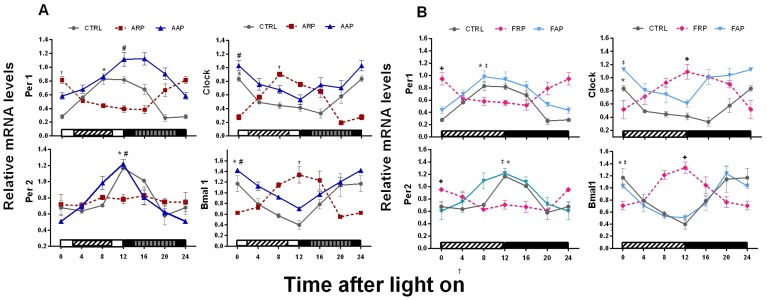
Except for Per2 in ARP and FRP, clock genes in the liver follow food and activity. Relative mRNA temporal profiles of clock genes along the light/dark (LD) cycle were examined in the liver of control. (n = 3 rats per time point). A) Black and white bars at the bottom of the figure represent the LD cycle; the striped bars represent the time spent in the slow rotating wheel for the ARP an AAP rats respectively. B) The striped bar indicates the light period in which FRP animals received their food. Quantification of genes was normalized to Actin. Daily curves for clock and metabolic genes were tested with a cosinor analysis to determine significance in amplitude and to determine the acrophase. See also table 2 for details. Asterisk indicates a significant rhythm in the CTRL group, † indicates a significant rhythm in the ARP group, # indicates a significant rhythm in the FRP group,+indicates a significant rhythm in the AAP group and ‡ indicates significant rhythm in the FAP group. These results were corroborated with Kruskall-Wallis test (Table 3).

In Control rats, mRNA of *Per1, Per2, Bmal1* and *Clock* in the liver revealed significant 24 h daily rhythms with high levels during the light-dark transition for *Per1* and *Per2* and in the transition dark-light for *Bmal1* and *Clock* (Figure 5A, table 2). AAP rats exhibited similar daily rhythms with slight increase in the mean values for *Clock* and *Bmal1* and with a significant shift towards the active phase for *Per1* (−3.6 h) (Figure 5A, Table 2). In contrast, ARP rats, showed an inversion in the rhythm of *Clock, Bmal1* and *Per1*, and the transcription of *Per2* was arrhythmic. In rats exposed to food during the active period (FAP), the temporal patterns of clock genes were similar as CTRL with exception of Clock that exhibited a 2.7 h phase advance (Figure 5B, table 2). Similar to the ARP rats, the FRP group showed inverted temporal patterns of *Per1, Clock and Bmal1* and no significant amplitude indicating loss of rhythmicity of *Per2* in the liver (Figure 5B and Table 2, 3).

**Table 2:** Cosinor analysis of clock and metabolic gene expression.

			ACROPHASEIN ZT			DIFERENCESOFACROPHASES						AMPLITUDIN %		
	*CONTROL*	*ARP*	*AAP*	*FRP*	*FAP*	*ARP*	*AAP*	*FRP*	*FAP*	*CONTROL*	*ARP*	*AAP*	*FRP*	*FAP*
***Per1***	10.4	**23.5**	**14.0**	**23.5**	10.7	−**13.1**	−**3.6**	−**13.1**	−0.3	0.5	4.0	0.3	0.3	0.4
***Per2***	13.1	*	**11.1**	*	11.8	*	**2.0**	*	1.3	0.4	–	0.4	–	0.3
***Clock***	0.18/24.18	**9.9**	23.8	**13.2**	**21.5**	−**9.7**	0.4	**11.0**	**2.7**	0.4	0.5	0.3	0.3	0.2
***Bmal1***	22.3	**11.8**	23.6	**11.4**	21.5	**10.6**	−1.2	**11.0**	0.8	0.5	0.4	0.3	0.3	0.5
***Nampt***	15.0	*	13.4	*	**8.7**	*	1.7	*	**6.3**	0.3	–	0.3	–	0.3
***NAD***	13.3	*	12.2	*	**10.7**	*	1.2	*	**2.7**	0.3	–	0.3	–	0.2
***Pgc1***	11.2	**7.7**	**15.4**	**8.7**	**15.4**	**3.5**	−**4.2**	**2.5**	−**4.3**	0.7	0.3	0.4	0.2	0.3
***Ppar*** **α**	14.0	*	**11.9**	**23.4**	**11.1**	*	**2.2**	−**9.3**	**2.9**	0.3	–	0.3	0.2	0.4
***Ppar*** **γ**	7.9	*	**10.0**	*	**11.4**	*	−**2.1**	*	−**3.5**	0.3	–	0.4	–	0.3
***Sirt1***	–	**–**	–	–	–	–	–	–	–	–	–	–	–	–
***SIRT1***	–	**–**	–	–	–	–	–	–	–	–	–	–	–	–

P>0.05 was considered not rhythmic.

**Table 3:** Kruskall-Wallis test. P>0.05 was considered not rhythmic.

Groups/genes	PER1	PER2	CLOCK	BMAL1	Nampt	NAD+	*SIRT1*	SIRT1 PROT	PGC1α	Pparα	Pparχ
**CONTROL**	0.017	0.025	0.047	0.017	0.021	0.009	**NS**	**NS**	0.036	0.019	0.038
**AAP**	0.028	0.031	0.021	0.025	0.036	0.001	**NS**	**NS**	0.018	0.036	0.021
**ARP**	0.043	**NS**	0.009	0.012	**NS**	**NS**	**NS**	**NS**	**NS**	0.037	**NS**
**FAP**	0.008	0.036	0.041	0.04	0.015	0.025	**NS**	**NS**	0.024	0.033	0.039
**FRP**	0.032	0.049	0.032	0.045	**NS**	**NS**	**NS**	**NS**	0.016	0.034	0.014

### Shift Work or Food in the Rest Phase Alters Clock Controlled Genes in the Liver

**Figure 6:**
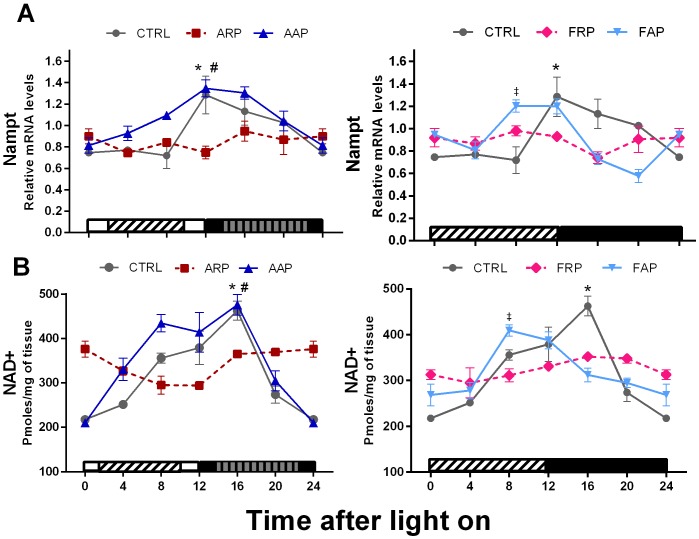
Nampt and NAD+ lose their rhythmicity in rats eating during the rest phase. Relative expression levels of Nampt mRNA,and NAD+ mRNA in liver of CTRL, ARP, FRP, AAP and FAP rats during the 24-h LD cycle. Each value represents the mean ± SEM (n = 3–4 per time point). Other indications as in Fig. 5.

Using a bioassay NAD^+^ levels were analyzed, whereas the levels of Nicotinamide phosphoribosyl transferase (*Nampt*) (which is an enzyme in the NAD+ salvage pathway) were measured by mRNA analysis. The expression levels of *Nampt* in CTRL animals showed a circadian rhythm with high values at the beginning of the dark period (acrophase at ZT13.3). A similar temporal pattern was observed in AAP rats, exhibiting a phase advance of 1.7, whereas the FAP rats exhibited a phase advance of 6 h according to the cosinor analysis (Figure 6, Table 2). Rats exposed to ARP and FRP protocols showed a loss of *Nampt* daily rhythm and their mean expression was of a significant lower level than CTRL rats. CTRL and AAP animals showed a significant rhythm of NAD^+^, with the highest values in the first half of the active phase. In agreement for what has been observed for *Nampt,* the ARP and FRP animals exhibited low amplitude of NAD^+^ resulting in loss of rhythmicity, whereas AAP and FAP animals showed a significant rhythm of NAD^+^ with a respectively 1.2 and 2.7 h advance as compared to CTRL (Figure 6 and Table 2,3).

**Figure 7:**
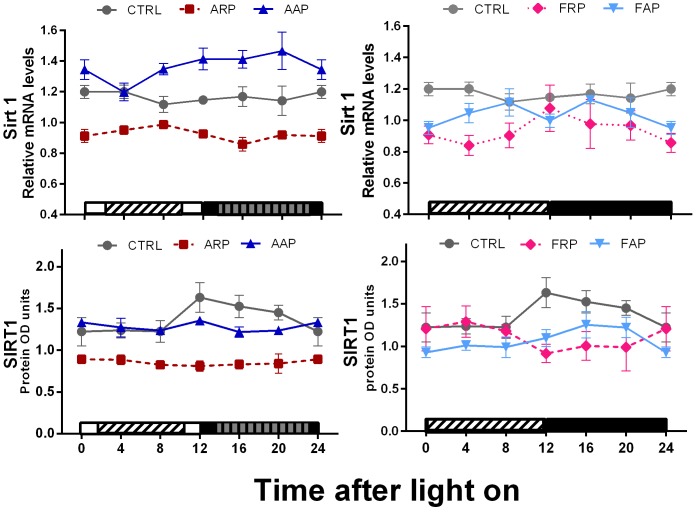
When rats are active and eat during the rest phase metabolic genes are down regulated. Relative expression levels of Sirt1 mRNA and SIRT1 protein in liver of CTRL, ARP, FRP, AAP and FAP rats are presented during the 24-h LD cycle. Each value represents the mean ± SEM (n = 3–4 per time point). Other indications as in Fig. 5.

None of the groups exhibited a significant daily rhythm of *Sirt1* mRNA or its protein SIRT1 (Figure 7, table 2). The main effect observed in ARP and FRP animals was a 25–30% decrease in *Sirt1* mRNA and protein, as compared with the CTRL and the AAP group showed an increase in *Sirt1* mRNA.

**Figure 8:**
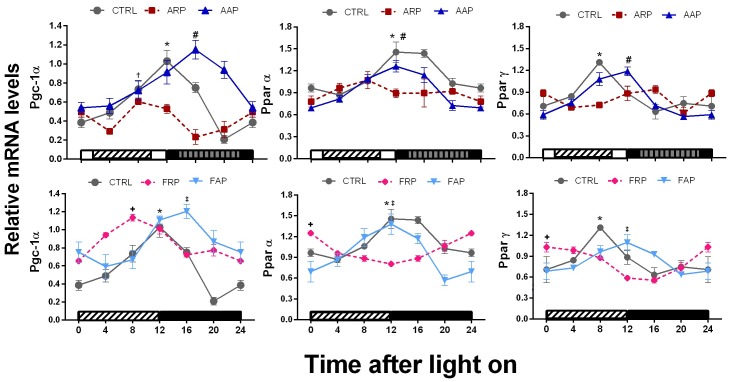
PGC-1α and Ppar genes show a loss of rhythm and are down regulated when rats eat during the rest phase. Relative expression levels of Pgc-1α, Pparα and Pparγ mRNA in liver of CTRL, ARP, FRP, AAP and FAP rats, during the 24-h LD cycle (n = 3–4 per time point). Each value represents the mean ± SEM. Other indications as in Fig. 5.

Hepatic nuclear receptors and transcriptional co-activators involved in the regulation of liver gluconeogenesis and fatty acid oxidation, showed *c*lear daily rhythms in *Pgc1α, Pparα and Pparγ* in the CTRL rats with peaks in the day-night transition and in the second part of the day for *Pparγ*. In the AAP and FAP groups daily rhythms were observed with a shift towards the active phase for *Pgc1α* and *Pparγ* (−4.2 h and −2.1 respectively) (Figure 8 and table 2,3). In contrast, ARP and FRP rats showed a phase advance in the rhythm expression of *Pgc1α* towards the inactive phase (3.5 and 2.5 h respectively) (Table 2,3). In addition ARP rats showed a loss of rhythmicity of both Ppar’s. (Figures 8, and Table 2,3).

## Discussion

Several studies have demonstrated that the ingestion of food during the rest phase induces dissociation of the rhythmic processes in the liver as compared to the rhythm indicated by the SCN [24], [25]. However not every organ reacts in the same manner to food elicited signals; some follow more the signals of the SCN [25]. This process is called internal desynchronization and is assumed to be one of the main causes for metabolic disorders and associated diseases in human shift workers. In our experimental model, using rats that are forced to be active in their rest phase (ARP), we show that this induced activity, changes the normal food intake to the rest period as it is indicated by the SCN. In this paradigm we focused on changes of clock and metabolic gene expression in the liver because it is probably the most important organ in the regulation of metabolism and most fundamental studies on the interaction between clock genes and metabolic genes were executed on the liver. Here we show that voluntary food intake during activity in the rest phase induces a dissociation of the rhythm of the liver clock genes as compared to their normal rhythm. More importantly it causes a desynchronization in the liver between the rhythm of clock genes and the metabolic genes. In rats with food restricted during the inactive phase, similar effects occurred but of lower intensity, suggesting that the combination of activity and food during the inactive phase has more severe consequences for metabolism and liver synchrony. Moreover under the present desynchronized conditions it was observed that the dissociation of the rhythm of metabolic genes from the rhythm of clock genes was concomitant with a down regulation of metabolic genes in the liver and with a lower glucose tolerance and with anatomical features of liver steatosis. This is the first study showing the relationship between clock genes and metabolic genes in our shift-work model in addition because of the importance of NAMPT for the production of NAD and SIRT1 [26]; we focused in the present study on the relationship between clock gene expression and the NAMPT-NAD-SIRT1 cascade including the measurement of *Pparα*, Pparγ and *Pgc1α*. The surprising new observation of the present study is that NAMPT and NAD lost their coupling with the clock genes *Bmal1* and *Clock* which contradicts earlier studies where such relationship has been demonstrated.

### Shift Work or Food Intake during the Rest Phase Cause Metabolic Alterations

Shift work or food intake in the rest phase induces a circadian misalignment of clock and metabolic genes within the liver, a gain of body weight and adipose tissue accumulation. In an earlier study [23] we focused on the question whether activity or the combination of activity and food was the most disturbing. The results of that study clearly indicated that only when activity in the rest phase was combined with (voluntary) food intake the animals developed a disturbed metabolism. Therefore in the present study where the focus was to investigate to what extend this desynchronization protocol also could affect clock and metabolic gene expression the combination of activity and food manipulation was not considered, further experiments should clarify the effects of the different combinations of food and activity on clock and metabolic genes. The decreased glucose tolerance and the signs of liver steatosis suggest that uncoupling feeding-related processes from the light-dark cycle is deleterious for health. The fact that the total consumption of food was similar in all groups indicates that the main factor promoting obesity was the disturbance in food intake and metabolic rhythmicity inducing the flattening of rhythms and decreased expression of all metabolic genes measured. Moreover when animals were also active during their normal rest phase (ARP) an additional loss of *Pparγ* rhythm was noticed together with more severe signs of liver steatosis suggesting that activity itself could also have additional effects. Dissimilarity in energy expenditure between the different groups could in principle explain the disparity in accumulation of fat in the worker animals. However since food intake in all groups is the same and the FRP and certainly ARP animals are active both during the light and during the dark phase such explanation does not seem realistic future studies may be able to resolve this.

Our data confirm previous reports in rodents and humans indicating that eating during the normal resting phase leads to a loss of blood glucose rhythm and to overweight [22], [26]–[29].

Our data also agree with a recent study reporting that in a mouse model of sleep restriction a disruption of diurnal rhythms leads to changes in metabolic functions [30]. Moreover human data indicate that in night-active persons the low amplitude and desynchronous timing of endocrine and metabolic rhythms stem from shifted eating patterns [31], [32]. Similar to our rodent model, in human populations, shifting activity and the main food consumption toward the rest phase also results in propensity to obesity and increased accumulation of abdominal fat [27], [32]. Moreover, human studies on non-breakfast eaters and night-eating syndrome patients also indicate that the timing of food intake is a determining factor in metabolic syndrome and weight gain [27]. Hereby the increase of abdominal fat is considered a predisposing factor for metabolic syndrome, including diabetes and cardiovascular disease [32] however this can also be seen as a symptom of a disbalance in the output of the autonomic nervous system [4], [33].

### Clock Genes are Inverted by Food and Per2 Loses its Rhythm with Food and Activity

In FRP animals all clock genes were inverted which is in agreement with earlier studies showing a similar inversion in rhythmicity [24], [25]. Interestingly in the “shift working” animals (ARP) that consume the majority (but not all) of their food in the rest phase, instead of inverted, *Per2* expression was completely flattened indicating that the additional activity might be a factor that disturbs the rhythm of *Per2*. In mice it has been shown that especially *Per2* in the liver is responsive to changes in feeding schedules [25]; which may indicate that the small amount of food intake during the active phase in the ARP animals might be enough to flatten the *Per2* expression. At the other hand *Per2* in the liver is, also responsive to autonomic or hormonal signals driven by the SCN [25], [34] that may result in a conflict of signals in the ARP rats with a consequent loss of rhythmicity and a lower overall expression. Rats forced to be active in their normal activity phase (AAP) exhibited a similar rhythm of clock genes in the liver as compared to control *ad libitum* fed animals confirming that activity in the wheels becomes only a disruptive stimulus when scheduled at the wrong time and associated with food intake [23]. Since we have demonstrated that also in the dark period rats preferentially eat during the period spent in the activity wheels [22], the *Per1* phase delay in AAP rats as compared to CTR and FAP animals suggests a strong influence of activity combined with feeding time on *Per1*. In view of these selective changes in clock gene expression and the accumulating molecular evidence that indicates a tight link between the circadian clock, its clock gene machinery and energy metabolism [12], [13], [19], [35] we investigated the possible impact of this “night work” protocol on liver metabolic genes.

### Dissociation of the Rhythm of Metabolic Genes from Clock Genes by Shift Work or Food in the Rest Phase

Surprisingly, in spite of the data suggesting the direct production of NAMPT via CLOCK/BMAL1 [36], [37], we observed in ARP animals that NAD^+^ and *Nampt* together with the metabolic genes *Pgc-1α, Pparα,* and *Pparγ* did not follow the inversion of the rhythm in the core clock genes; they all lost their rhythm. The absence of such changes in the AAP rats and the observation that in FRP rats *Nampt* and NAD^+^ were also uncoupled from the rhythm of *Clock* and *Bmal1* indicates that mainly the change in food intake induced a desynchronization within the liver. The decrease of *Nampt* in the liver under ARP and FRP conditions agrees with the decrease of *Nampt* seen in *Clock* mutant mice [36] and might consequently also be related to the disturbed food intake of these *Clock* mutants. This study [36] also showed that inhibition of *Nampt* affects especially *Per2* levels possibly via the inhibition of SIRT1. The co-enzyme NAD+ together with its rate-limiting biosynthetic enzyme NAMPT stimulates the production of SIRT1 which is a protein that coordinates metabolic programs like gluconeogenesis, glycolysis and lipid metabolism [37]–[39] and has proposed a feedback role on clock genes by inhibiting *Clock* and *Bmal1* [10], [36]. The observation of a simultaneous decrease in SIRT1, together with the decrease and loss of rhythm in *Per2* and *Nampt*, suggests an important disturbance of these three elements in the link between the core clock and metabolism, in circadian misaligned animals. The uncoupling of the rhythm of *Nampt* from that of *Clock* and *Bmal1* raises the question which other cellular messengers will form the link between food (i.e. glucose uptake), metabolic genes and clock genes and demonstrate the necessity of using physiological models in order to understand and validate proposed interactions of the molecular networks in the cell. In this respect it might be worth to consider that peroxiredoxins which are evolutionary extremely conserved molecules associated with the oxidative state of the cell and show a rhythm in their oxidation level which is associated with the metabolic cycle may influence the synthesis of NAMPT [40].

Previous observations indicated that rats without SIRT1 develop liver steatosis while mice that over express SIRT1 are protected against diet induced liver steatosis [14], [15]. The low levels of SIRT1 observed in ARP rats are in agreement with their propensity to accumulate abdominal fat and their significant overweight. Several studies show SIRT1 as an important regulator stimulating the transcriptional activity of *Pgc1α, Pparα and Pparγ* [14], [41]–[46], we suggest that the consequence of low *SIRT1* and *Pgc-1α* together with the decrease of the PPAR’s in ARP rats may lead to overweight and metabolic disturbances as observed for example in the *Sirt1* knockdown mice [14], [15], [43]–[44]. Moreover in line with these observations, it has been shown that an increase of SIRT1 improves insulin sensitivity [47] indicating that indeed the low levels of SIRT1 observed in the present study may reflect a state of lesser sensitivity for insulin. Surprisingly, *Pgc-1α* in FRP animals was not only shifted to the moment food was available it was also up regulated and did not resemble the ARP animals. It is possible that this additional effect may explain why the glucose changes and liver accumulation of fat is more pronounced in the ARP animals. In spite of several studies that show that the nuclear receptor genes like *Pparα* and *Pparγ* are (in)direct targets of circadian clock genes [16], [44], [45], the present study illustrates that metabolic genes such as *Nampt*, *Sirt1* and *Pparα,* may have additional regulation mechanisms not linked with the core clock genes.

Taken together, the present study demonstrates that feeding and forced activity in the rest phase not only desynchronizes the rhythm between the SCN and the liver but also disturbs internal molecular rhythms within the liver, especially the relationship between the clock genes and the metabolic genes. This may explain why “night-work” results in diseases associated with metabolic disorders such as obesity and diabetes by down regulating metabolic genes such as *Nampt*, *Sirt1, Pgc-1α* and *Pparα*. This highlights the complexity of the interaction between the circadian system and metabolism, and poses a daunting challenge to understand the circadian metabolic network that has been shown in the present study to be so extremely vulnerable for a temporal disturbance in the balance of its components.
